# CAREGIVER TLC: A VIRTUAL PSYCHOEDUCATIONAL PROGRAM FOR CAREGIVERS: BASELINE PRELIMINARY DATA

**DOI:** 10.1093/geroni/igac059.2558

**Published:** 2022-12-20

**Authors:** Julian Montoro-Rodriguez, Dolores Gallagher-Thompson, Jennifer Ramsey, Ann Choryan Bilbrey, Bruno Kajiyama, Kendra Jason, Larry W Thompson

**Affiliations:** University of North Carolina at Charlotte, Charlotte, North Carolina, United States; Stanford University, Stanford, California, United States; Case Western Reserve University, Cleveland, Ohio, United States; Optimal Aging Center, Sunnyvale, California, United States; Photozig, Inc., Moffett Field, California, United States; University of North Carolina at Charlotte, Charlotte, North Carolina, United States; Optimal Aging Center, Sunnyvale, California, United States

## Abstract

Family caregivers are at increased risk for negative impacts on their psychological and physical health compared to non-caregivers. Virtual caregiving programs are beneficial as caregivers may not have time to devote to face-to-face programs and especially important to caregivers in the context of the COVID-19 pandemic. Our team at the University of North Carolina at Charlotte is testing the efficacy of the Caregiver Thrive, Learn & Connect Virtual Program adapted from the Coping with Caregiving evidence-based multicomponent intervention (Gallagher-Thompson et al., 2003). The program offers to registered caregivers six weekly sessions over Zoom teleconferencing in small groups led by trained professionals from community partners serving socio-demographically diverse caregivers. Sessions address stress management, mood management, resilience, self-care, coping strategies, and isolation. Preliminary baseline data on 42 participants indicates that caregivers are primarily female (87%), on average 64 years old, and from diverse racial backgrounds: white (69%), African American (29%) and Asian American (2%). Participants provide care to persons with memory troubles or dementia (66%) and chronic health conditions (34%). Baseline data on initial levels of caregivers’ psychosocial outcomes indicated salient levels of mental health outcomes for burden (high = 49%; mild = 35%); anxiety (moderate = 16%; severe = 20%) and depression (mild levels = 35%; moderately and severe level of depression = 33%). Caregivers for chronic health conditions reported significantly higher anxiety compared to dementia caregivers. The Caregiver TLC program offers support to the targeted caregiver population seeking to improve their level of competence, mental health and social isolation.

